# Data Demonstrating a Lack of Black and White Parity in Breast Cancer Mortality Following Medicaid Expansion: Are the Data Granular Enough to Support This?

**DOI:** 10.1200/GO.20.00428

**Published:** 2021-02-02

**Authors:** Quyen D. Chu, John F. Gibbs, John M. Lyons, Tingting Li, Mei-Chin Hsieh, Yong Yi, Xiao-Cheng Wu

**Affiliations:** Quyen D. Chu, MD, MBA, Department of Surgery, LSU Health Sciences Center-Shreveport, Shreveport, LA; John F. Gibbs, MD, Hackensack Meridian School of Medicine, Nutley, NJ; John M. Lyons, MD, Our Lady of the Lake Regional Medical Center at Baton Rouge, Baton Rouge, Louisiana; and Tingting Li, MPH; Mei-Chin Hsieh, PhD; Yong Yi, PhD; and Xiao-Cheng Wu, MD, MPH, Louisiana Tumor Registry & Epidemiology and School of Public Health at LSU Health Sciences-New Orleans, New Orleans, LA

We read the study by Semprini and Olopade^1^ with great interest. In their difference-in-difference analysis of the impact of Medicaid expansion on Black and White breast cancer mortality, the authors found Black women to have a higher mortality rate than White women. These findings run counter to other studies that demonstrated a positive effect of the Affordable Care Act (ACA) Medicaid expansion.^2,3^

We believe that Semprini and Olopade's^1^ study suffers a number of shortcomings. Their study represents a snapshot of a bird's eye view of the ACA Medicaid expansion landscape. It is devoid of granular data, which are critical to understand the intricacies of ACA expansion. What was the proportion of early-stage and late-stage diseases between the Black and White cohorts? If more Black women present with higher stage disease than White women at the time of Medicaid enrollment, naturally, one would expect a higher mortality rate.

What were the comorbidities of the cohorts? Were the authors' analysis adjusted for other covariates such as tumor, treatment, and social determinant of health (SDoH)? In fact, emerging data strongly support a link between cancer outcomes and SDoH, which describes a complex set of conditions in which people are born, grow, live, work, and age and that these circumstances are shaped by the distribution of money, power, and resources at global, national, and local levels.^4^ Why is this important? Because optimal treatment such as surgery and chemoradiation therapy accounts for only 10%-20% of the variation in premature mortality^5^; the remaining 80%-90% is dependent on SDoH such as safe housing, access to education and health services, and availability of healthy foods.^6,7^ Is it possible that, everything being equal, the higher mortality observed in a Black woman has nothing to do with ACA expansion, but has everything to do with her zip code? If she lives in a food desert neighborhood or lacks the necessities to keep her safe, can ACA expansion be culpable for her mortality? ACA expansion cannot be the panacea for societal inequities.

The authors' study relies on a Black/White mortality ratio. It is theoretically plausible that the mortality could have improved in both White and Black patients, but the ratio might have missed them. Perhaps, a much better metric to use would have been to assess the absolute mortality reduction for each race or ethnicity. Additionally, there is no control for the potentially lower power in the younger age groups or in states that may have a small number of Black residents.

As alluded to by the authors, longitudinal data are needed for reliable mortality analysis. We know from large meta-analyses that treatments in breast cancer may demonstrate an early benefit in reducing cancer recurrence, but improving survival and reducing mortality often takes 5, 10, and sometimes 15 years (as is the case with radiation therapy) to realize.^8^ Therefore, assessing the success or failure of Medicaid expansion solely by using 2- to 3-year mortality rate in a disease like breast cancer may not be a reliable metric.

We concur with the authors' comment that a “robust analysis of specific states or healthcare systems” is needed. We recently evaluated the impact of ACA Medicaid expansion on 14,640 Louisiana women with stage 0-4 breast cancer and found that the expansion significantly reduced the uninsured rate by almost 50%, increased the proportion of patients diagnosed with early-stage disease by 27%, increased the proportion of patients receiving postoperative radiation following breast-conserving surgery by 19%, and decreased the proportion of patients having delayed receipt of radiation for early-stage disease by 16%.^3^

Our concern is that the authors' study further obfuscates the debate surrounding ACA expansion. As of May 2020, 37 states, including the District of Columbia, have adopted ACA expansion, whereas 14 states have not, many of which are located in the south, which is home to nearly half of the nation's uninsured population before the ACA.^9^ Compared with nearby states, Louisiana has the lowest uninsured rate (8%); Arkansas, which is an ACA expanded state, has an uninsured rate of 8.23%, whereas Texas, Oklahoma, and Mississippi, which are non-ACA expanded states, have uninsured rates of 17.71%, 14.20%, and 12.13%, respectively.^10^ Our report underscores the importance of states or healthcare systems in obtaining granular data that are specific to their health organizations. We, therefore, recommend that readers should view the study by Semprini and Olopade^1^ with a full understanding of its limitations.
